# Dietary supplementation with phytosterol and ascorbic acid reduces body mass accumulation and alters food transit time in a diet-induced obesity mouse model

**DOI:** 10.1186/1476-511X-10-107

**Published:** 2011-06-28

**Authors:** Sheila J Thornton, Ian TY Wong, Rachel Neumann, Petri Kozlowski, Kishor M Wasan

**Affiliations:** 1 Faculty of Pharmaceutical Sciences, University of British Columbia, 2146 East Mall, Vancouver, BC, Canada

**Keywords:** Obesity, phytosterols, phytostanols, ascorbic acid

## Abstract

Previous research indicates that animals fed a high fat (HF) diet supplemented with disodium ascorbyl phytostanyl phosphate (DAPP) exhibit reduced mass accumulation when compared to HF control. This compound is a water-soluble phytostanol ester and consists of a hydrophobic plant stanol covalently bonded to ascorbic acid (Vitamin C). To provide insight into the mechanism of this response, we examined the *in vivo *effects of a high fat diet supplemented with ascorbic acid (AA) in the presence and absence of unesterified phytosterols (PS), and set out to establish whether the supplements have a synergistic effect in a diet-induced obesity mouse model. Our data indicate that HF diet supplementation with a combination of 1% w/w phytosterol and 1% w/w ascorbic acid results in reduced mass accumulation, with mean differences in absolute mass between PSAA and HF control of 10.05%; and differences in mass accumulation of 21.6% (i.e. the PSAA group gained on average 21% less mass each week from weeks 7-12 than the HF control group). In our previous study, the absolute mass difference between the 2% DAPP and HF control was 41%, while the mean difference in mass accumulation between the two groups for weeks 7-12 was 67.9%. Mass loss was not observed in animals supplemented with PS or AA alone. These data suggest that the supplements are synergistic with respect to mass accumulation, and the esterification of the compounds further potentiates the response. Our data also indicate that chronic administration of PS, both in the presence and absence of AA, results in changes to fecal output and food transit time, providing insight into the possibility of long-term changes in intestinal function related to PS supplementation.

## Introduction

Obesity is characterized by an over-accumulation of adipose tissue that is normally associated with excess caloric intake. Over the last few decades, research in the field of obesity has lead us far from the concept of adipose tissue as an energy storage depot. We now understand that fat is a secretory tissue, producing a variety of bioactive substances collectively referred to as adipocytokines. A number of these substances, notably tumor necrosis factor (TNF) alpha and plasminogen activator inhibitor type 1, suggest that obesity is essentially an inflammatory disease, where excess adipose tissue induces macrophage production and activates the immune system without a legitimate pathogen [1]. Numerous studies indicate that the dysregulation of these adipocytokines may directly contribute to obesity-related diseases [2]. In addition to adipocytokines, the production of reactive oxygen species (ROS) has also been shown to be elevated in obesity and diabetes [3,4]. Hyperglycemia, hyperlipidemia and hypercholesterolemia, key clinical manifestations of obesity and diabetes, all promote ROS production through various pathways [5].

Plant sterols (phytosterols) and their saturated derivatives, phytostanols, are among a growing list of dietary components that exert a positive effect on hypercholesterolemia. It is well established that certain plant sterols and stanols reduce plasma cholesterol levels, ostensibly by inhibiting enterocytic cholesterol uptake through competition with dietary and biliary cholesterol for absorption [6-8]. The modification of hydrophobic plant stanols into a water-soluble phytostanol ester, where the phytostanols were covalently bonded to ascorbic acid (Vitamin C), was undertaken to combine the hypocholesterolemic properties of the phytostanol with the potential benefits of an antioxidant [9,10]. The resulting chemical, disodium ascorbyl phytostanyl phosphate (DAPP) is a phytostanol analogue that exhibits a more potent hypocholesterolemic effect than unesterified phytosterols and stanols alone [11-14].

Data from studies examining the hypocholesterolemic effects of DAPP also indicated that animals treated with the compound experienced a dose-dependent decrease in body mass accumulation [11,15]. Further analysis of this compound-associated mass loss revealed that adipose tissue stores were reduced with no accompanying reduction in lean body mass [16,17]. When previously obese animals were treated with 2% w/w dietary disodium ascorbyl phytostanyl phosphate in addition to their high fat diet (45% kcal from fat), they immediately began losing adipose tissue and within 8 days, they achieved a statistically similar body mass to untreated animals on a low fat diet (10% kcal from fat). After 60 days of compound administration, treated animals exhibited a 41% decline in total body fat with no adverse effects on organ mass, femur length, muscle mass or gross morphology; in addition, a significant increase in aerobic scope (VO_2swim _- resting metabolic rate) was observed. In effect, the compound turned previously obese mice on a high-fat diet into a low-fat diet phenotype [17].

Ingestion of unesterified plant sterols has been reported to result in loss of adipose stores [18]. Research also indicates that mice on a high fat diet supplemented with high doses of ascorbic acid accumulate significantly less adipose tissue than their non-supplemented counterparts [19-22]. By examining the *in vivo *effects of diet supplemented with ascorbic acid in the presence and absence of phytosterols, we set out to establish whether the supplements have a synergistic effect in a diet-induced obesity mouse model, and to investigate possible mechanisms for the supplement-induced mass loss.

## Materials and methods

### Animals and Diets

All animal studies were conducted with approval from the UBC Animal Care Committee. Male C57BL/6 mice (4 weeks old) were purchased from Charles River laboratories (St. Constant, Quebec, Canada) and housed individually with wood shaving bedding. The mice were kept at a constant temperature of 21°C ± 2°C in a 12 hr light/dark cycle and had unrestricted access to food and water throughout the period of the study. Animal mass, food mass and water intake were recorded once per week. Metabolic assessment of all animals was initiated on week 13; therefore only data from the first twelve weeks was used for mass accumulation analysis.

Following an acclimatization period of eight days on regular mouse chow, C57Bl/6 mice were randomly assigned into 4 groups (*n *= 8) and placed on the diets for 18 weeks. Using Research Diet's 45 kcal% fat diet D12451 as a high fat control (HF), diet supplements were milled into the HF control diet using either 1% w/w phytosterol/stanol mixture (85451; Sigma Aldrich, Oakville, ON; β-sitosterol ~76%, sitostanol ~13%, campesterol ~8%, campestanol ~1%) for the PS diet, 1% w/w L-ascorbic acid (A5960; Sigma Aldrich, Oakville, ON) for the AA diet, or a combination of 1% phytosterol/stanols and 1% L-ascorbic acid for the PSAA diet.

### Oxygen Consumption

Animals were evaluated for whole animal metabolic consumption using indirect flow-through calorimetry during weeks 13-15. Measurement of oxygen consumption at rest (resting metabolic rate; RMR), and at maximal swimming rate (VO_2swim_); were conducted on each dietary group.

Resting metabolic rate is defined as the lowest average oxygen consumption at 21 ± 0.2°C over a 5 min period during the light phase (between 1000 and 1800 h) using an open flow respirometry system. For RMR assessment, animals were placed in a sealed black Plexiglas 1075 ml chamber immersed in a 21 ± 0.2°C water bath as per methodology outlined in Thornton et al, 2007 [17]. Briefly, outside atmospheric air was pushed through the chamber at a rate of 800 ml min^-1 ^(0-1.5 l air pump, Rena Air 400A; Aalborg Mass Flow controller (0-5 l)). A subsample of excurrent air was dried and scrubbed of CO_2 _and passed through an oxygen analyser (Beckman OM-11 polarographic oxygen analyzer) at a rate of 300 ml min^-1^. Oxygen measurements were recorded each second via a DI-710 A/D converter and the lowest 5 min was corrected for pressure and temperature, and then averaged to estimate RMR (Windaq DATAQ software). Immediately prior to all metabolic measurements, body mass was recorded to ± 0.1 g. Mice were not denied food or water prior to respirometry measurements; however, as most food intake occurs nocturnally, it is an accepted practice to assume the animal is approaching a post-prandial state near the end of a RMR assessment [23,24]. Each animal remained in the chamber for a minimum of 2 hours to ensure that RMR had been achieved.

To measure VO_2swim_, a glass funnel was suspended over a water bath maintained at 20 ± 0.2°C. The animals were introduced into the water bath and the funnel was submerged over the mouse to a predetermined height, leaving an air volume of 250 ml above the water level. Room air was introduced into the funnel at a rate of 800 ml/min via a submerged air stone. The air stone was positioned directly beneath the animal to encourage active swimming. VO_2swim _was defined as the highest oxygen consumption averaged over 2 min of a 5 min swim trial. A subsample of excurrent air was evaluated as described above.

### Body composition

Whole body fat measurements were carried out on a 7T animal MRI scanner (Bruker, Germany) on week 16 of the study. Unanesthetised mice were placed inside a Plexiglas restrainer, and the restrainer was positioned inside the bore of the magnet. NMR signal from the entire body was acquired with a quadrature volume RF coil tuned to 300MHz. A standard CPMG sequence (TE = 2.377 ms, TR = 10 s) was used to acquire 256 echoes from which the T2 decay curve was extracted. The decay curves were then fit to a double exponential function using software procedure developed in house with Igor (WaveMetrics, OR). The ratio of lean tissue/body fat expressed as weight/weight is then calculated from the NMR data as described previously [25].

### Food transit time

Food transit time was measured by timing the fecal appearance of Sudan Red III dye administered in the diet as per Toloza et al, 1991 [26]. Briefly, 300 mg Sudan red III dye was dissolved in 250 mL of acetone, and 300 mg of each diet was placed in a glass beaker and covered with the Sudan red/acetone mixture. The dyed rations were placed in a fume hood for 72 hours of evaporation to eliminate the acetone.

To assess the effect of chronic exposure to a HF diet with or without supplements on food transit time, on week 18 of the experiment, all mice were switched to the non-additive HF control diet for 24 hours. At 5 am, animals were offered the HF control Sudan Red III dyed diets for two hours, followed by a collection of feces for twenty hours during *ad libidum *feeding of HF control ration. Fecal samples from each collection time point were dried for a minimum of 48 hours at 60°C and stored at -20°C until analysis. Fecal pellets were ground using a mortar and pestle and a ~100 mg aliquot was placed in a screw top test tube, vortexed with acetone in a 50:1 w/v ratio and allowed to stand at RT for 30 min. The samples were centrifuged at 3000 rpm for 3 min and the quantity of dye in the acetone was assessed spectrophotometrically at 504 nm. The total amount of dye eliminated by each mouse during the 20 h collection period was taken to be 100%, and the data from each time point are expressed as a percent excretion of the total dye released over the twenty-hour collection period. Curves were then fit to a sigmoidal logistic function using Systat and individual times for 10% through 100% dye excretion were calculated. Values were indexed to diet and analysed using a one-way ANOVA; significantly different group means were identified using Tukey Test.

To establish the acute effect of dietary components on food transit time, age-matched C57Bl/6 mice raised on normal chow were randomly assigned a dietary group and were exposed to the experimental diet for 72 hours prior to food transit time analysis using above methodology.

### Digestive efficiency

Digestive efficiency is a measure of the amount of chemical energy absorbed from the diet. As the diets have slight differences in caloric value and sterol content, the values of fecal energy outputs were corrected for consumed phytosterol/stanol amounts. Diets were relatively isocaloric between the HF control (4.73 kcal/g), AA (4.69 kcal/g), PS (4.68 kcal/g) and PSAA (4.65 kcal/g) rations. As it is assumed that phytosterols are not absorbed, the caloric content of PS is not included in the above caloric content. Feces were collected over a period of 72 hr by replacing the shavings in each cage with a metal grid to facilitate fecal collection. Collected fecal material was dried overnight in an oven at 60°C, then stored at -20°C until analysis. Fecal energy content was assessed using a bomb calorimeter (Adiabatic calorimeter 1241, Parr Instrument) and corrected for phytosterol content. Digestive efficiency was calculated for each animal as the difference between gross energy consumed and fecal energy output over a 72-hour period.

### Statistical analysis

Statistical analysis was conducted using Sigmastat (Systat for Windows; v. 5.02). To test effects of diet and supplement, we used a one-way ANOVA; significantly different group means were then separated using a Tukey Test or Dunnett's Method as appropriate. Using Sigmaplot, passage rate analysis was conducted by curve fitting the data from each individual animal and calculating individual output values for 10-100% dye excretion. Values were indexed to diet and analysed using a one-way ANOVA in Sigmastat; significantly different group means were identified using an ANOVA and a post-hoc Tukey Test; observed differences between supplement groups and high fat control were reported. Values with *p *< 0.05 were considered to be significant.

## Results

### Growth curves

**Figure 1:**
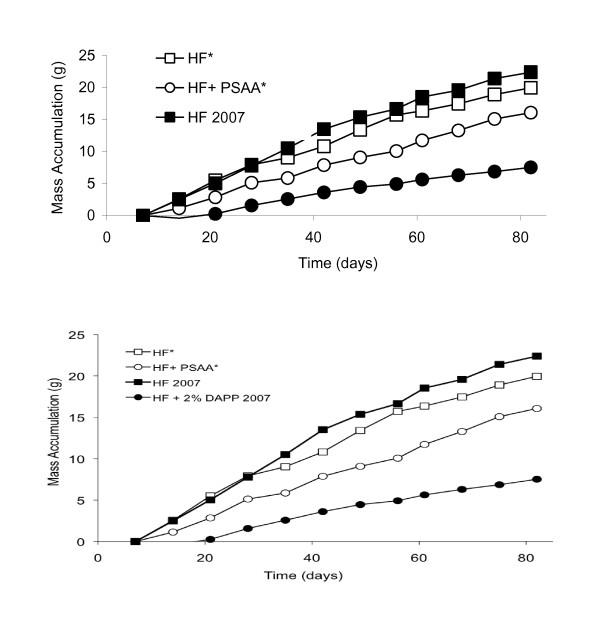
**Mass accumulation of male C57bBl/6 mice exposed to a high fat diet with or without supplements**. 1a) Animals in the phytosterol+ascorbic acid (PSAA) group exhibited a reduction in mass accumulation by week 2 and the difference became statistically significant by week 7 of the study (n = 7; ANOVA p < 0.05). 1b) Mass accumulation over time for C57Bl/6 animals consuming a high fat diet with ascorbic acid and phytosterol supplementation (this study) in comparison to animals consuming a high fat control diet with or without disodium ascorbyl phytostanyl phosphate^(17)^. Mice chronically exposed to a high fat diet supplemented with 2% w/w phytostanol esterified with ascorbic acid show a greater decrease in mass accumulation than those consuming a high fat diet supplemented with unesterified phytosterols and ascorbic acid (1% w/w of each component). * indicates data obtained from this study.

Over the first 12 weeks of the study, animals fed a HF diet supplemented with a combination of AA and PS exhibited a decrease in mass accumulation when compared to control (PSAA group, n = 8, ANOVA followed by a post-hoc Tukey; p < 0.05, Figure 1a). Animals in the PSAA group exhibited a lower mass by week 2 and this difference became significant by week 7 of the study (n = 7; p < 0.05). There was no significant difference in caloric intake or water intake between the dietary groups throughout the growth assessment period (12 weeks; ANOVA, data not shown).

### Oxygen Consumption

Metabolic assessment was conducted on animals during weeks 13-15 of chronic exposure to a HF diet with or without supplements. Dietary supplementation with PS, AA or PSAA did not result in significant differences of RMR or VO_2max _when compared with HF control (n = 8; ANOVA).

### Body Composition

**Figure 2:**
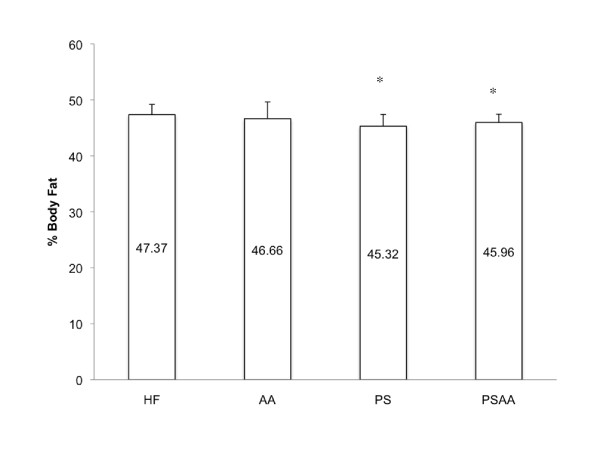
**Body fat significantly decreases with the addition of 1% phytosterol to a high fat diet when compared to HF control (HF n = 7, all other groups n = 8; ANOVA followed by a post-hoc Dunnett's Method)**. Animals were assessed using magnetic resonance imaging after 16 weeks on the dietary supplements.

The ratio of lean tissue/body fat expressed as weight/weight was measured for each animal using MRI. Small but significant differences were observed between the % body fat and lean-to-fat ratios in animals in the PS and PSAA groups when compared to HF control (n = 8; ANOVA, post-hoc Tukey; % body fat values HF 47.37%; AA 46.66%; PS 45.32%; PSAA 45.70%; Figure 2).

### Fecal Caloric Content

**Figure 3:**
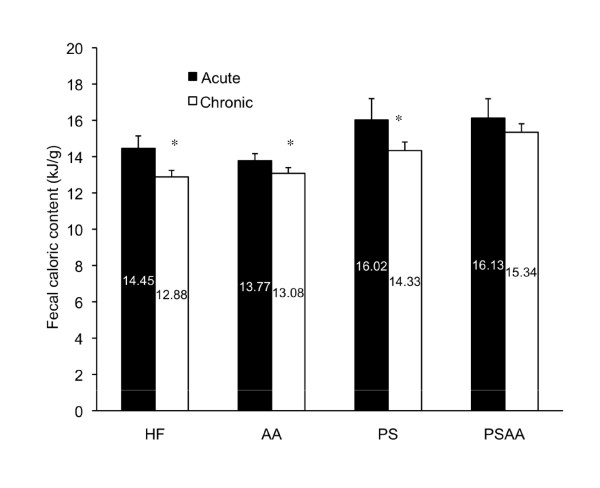
**Fecal caloric content in age-matched animals exposed to the diet for 72 hours (acute - closed bars) or 17 weeks (chronic - open bars)**. A significant decline in caloric content of the feces was observed in animals chronically exposed to the diet in the HF, AA and PS groups when compared to an acute 72-hour exposure in age-matched individuals (n = 7; t-test within each dietary group). Asterisks indicate a significant decline in fecal caloric content is observed in animals exposed to a diet for 17 weeks when compared to acute dietary exposure. After caloric content was corrected for estimated phytosterol content, no significant difference was observed between the diets within each exposure.

Fecal caloric content was compared both within the acute and chronic exposure groups as well as between each dietary group. After corrections were applied to account for fecal phytosterol content, we did not observe a significant difference between the caloric content of the feces between the diets in either the chronic or acute exposure groups (n = 8; ANOVA). When fecal caloric content was compared within each dietary group, we observed a significant reduction in fecal caloric content with chronic exposure to the HF (p = 0.0002), AA (p = 0.003) and PS (0.004) diets (n = 8; t-test). The PSAA group did not demonstrate a significant reduction in fecal caloric content after chronic exposure to the supplement (chronic 15.34 ± 0.47 vs acute 16.13 ± 1.06; p = 0.09; Figure 3).

### Food Transit Time

**Figure 4:**
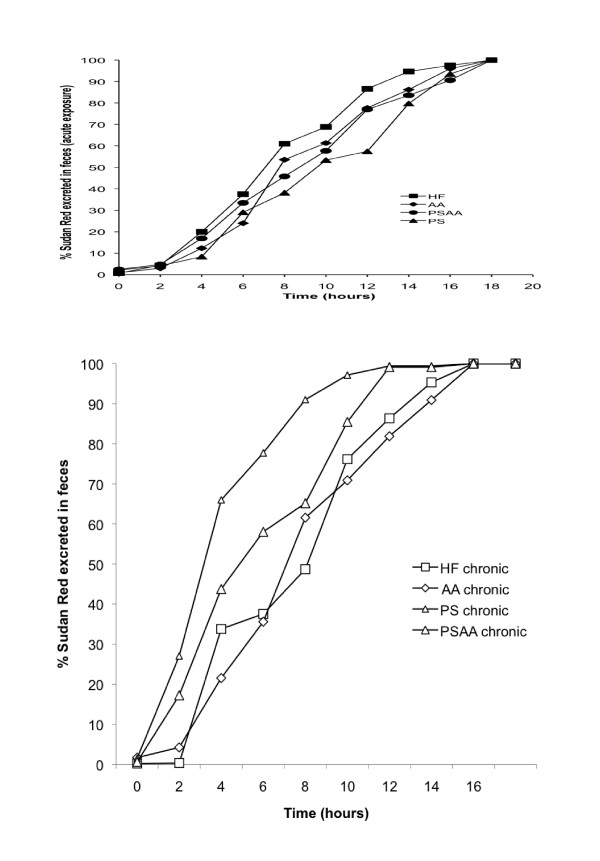
**Food transit time of age-matched C57Bl/6 mice after an acute (72-hour) and chronic (18 week) exposure to a high fat diet with or without ascorbic acid or phytosterol supplements**. 4a) Acute exposure to dietary supplements did not alter food transit time when compared to the high fat control (n = 7; ANOVA). 4b) Animals chronically exposed to PS and PSAA supplements exhibited significant differences in transit time of a HF meal when compared to the HF control (ANOVA with post hoc Tukey Test; n = 7 for HF and AA; n = 3 for PS, n = 4 for PSAA).

A comparison of the HF control group to the groups containing additives was undertaken for both the acute and chronic diet protocols in order to assess the effect of supplements on food transit time. Each dietary group was also statistically assessed to establish the effect of chronic exposure to the diet on food transit time. Spectrophotometric assessment of fecal dye content was expressed as % excretion over the 20-hour assessment period. Based on the assumption that dye consumption was complete at the midpoint of the 2-h pulse, dye recovery times for 10% through 100% excretion were calculated for each animal by curve fitting using a sigmoidal logistic function. Age-matched C57Bl/6 mice did not exhibit any differences in food transit time after an acute 72-hour exposure to a HF diet with supplements when compared to animals exposed to the HF control diet (n = 7, ANOVA; Figure 4a).

After 18 weeks of exposure to PS, AA or PSAA, dietary supplements were withdrawn for 72 hours in order to assess the chronic effect of additives on transit time. Placing all animals on the HF diet alone eliminated a possible intrinsic effect of the dietary supplements on passage rate. Animals in PS and PSAA exhibited significant differences in transit time when compared to the HF control (ANOVA with post hoc Tukey Test; Figure 4b). The 50% dye recovery values (mean ± SD) for each diet were eliminated at 9.00 ± 2.17 h (HF; n = 7); 8.39 ± 2.22 h (AA; n = 7); 5.2 ± 1.12 h (PS; n = 3), and 7.02 ± 2.47 (PSAA; n = 4). Some animals in the PS and PSAA groups did not consume any of the Sudan Red III dyed ration during the 2-h pulse, therefore the n number is lower in these groups.

**Figure 5:**
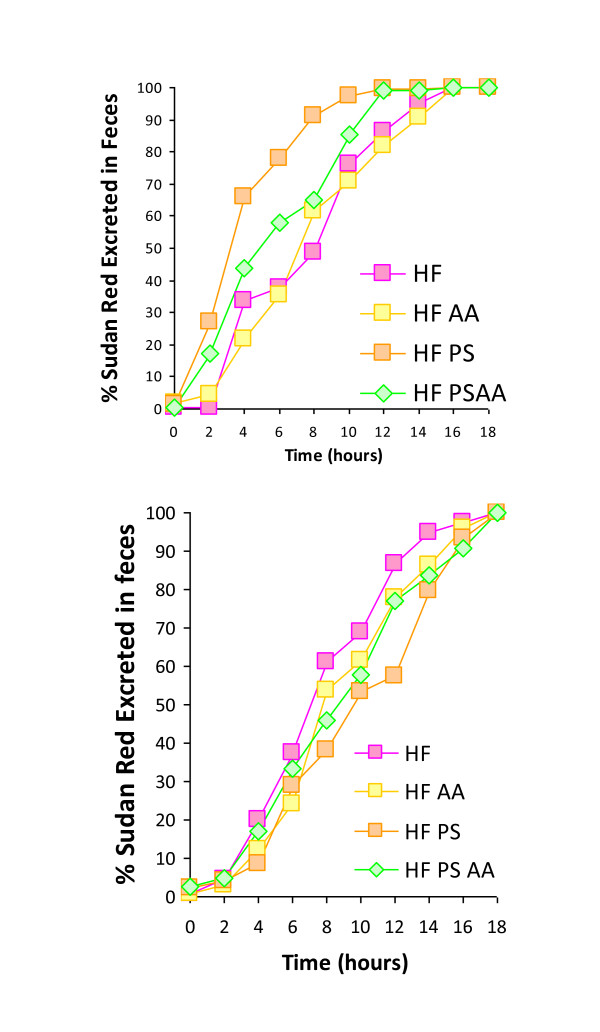
**Food transit time of a HF diet in animals chronically exposed to a high fat diet with or without ascorbic acid supplements is compared to acute (72 hr) exposure transit times**. 5a) In the HF and AA groups, chronic exposure to diets did not alter food transit time when compared to acute exposure to the diet for 72 hours in age-matched C57Bl/6 mice (n = 7; ANOVA followed by a post-hoc Tukey Test). 5b) Chronic exposure to a HF diet supplemented with phystosterol alone or phytosterol with ascorbic acid significantly reduced food transit time when compared to acute exposure to the diet for 72 hours in age-matched animals (n = 7; significant differences between PS chronic and acute were observed at 20 - 90%; PSAA chronic and acute at 80 and 90%; ANOVA followed by a post hoc Tukey Test).

When the acute food transit time was compared to chronic food transit time for each dietary group, no significant change in passage rate was observed for animals on the HF or AA diets (n = 7; ANOVA; Figure 5a). However, a significant decrease in food transit time was observed for animals in both the PS and PSAA supplemented diet groups (n = 3 and 4 respectively; ANOVA followed by a post-hoc Tukey Test, Figure 5b).

### Food consumption and fecal output

Weekly average food consumption and gross energy intake did not differ significantly between dietary groups (n = 8; ANOVA). Experimental manipulations (metabolic assessment, swim challenge, fecal collection grids) were associated with a temporary reduction of food intake; therefore, mass accumulation and food consumption data are reported only for the first 12 weeks of the study.

**Figure 6:**
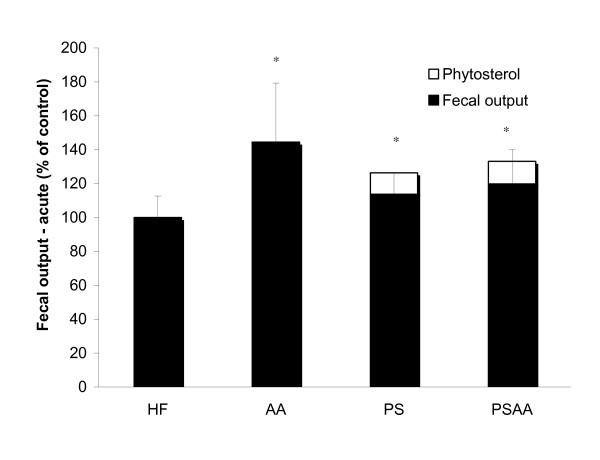
**Fecal output of age-matched mice after a 72-hour acute exposure to HF diets with or without supplement**. As phytosterols are assumed to be 100% excreted, fecal output for animals in the PS and PSAA groups were corrected to account for the 1% of dietary intake of non-digestible phytosterol (PS accounts for ~ 10% of fecal output, based on a daily consumption of 1% of 3 g chow and average daily fecal output of 300 mg). Acute exposure to all HF diets containing supplements resulted in a significant increase in fecal output when compared to HF control (n = 7; ANOVA followed by a post-hoc Dunnett's Method).

Fecal output was assessed under three different conditions. Acute exposure to HF diet with or without supplements in age-matched C57Bl/6 mice indicated that all diets containing supplements resulted in a significant increase in fecal output when compared to the HF control (n = 7; ANOVA followed by a post-hoc Dunnett's Method; Figure 6). In animals fed a 1% w/w phytosterol dietary supplement, a correction factor accounting for the undigested phytosterol component is applied. Based on the mean daily food consumption and fecal output (~3 g and 300 mg, respectively) the fecal mass in animals consuming a diet containing phytosterol is expected to be to be ~10% higher due to the non-digestible component.

**Figure 7:**
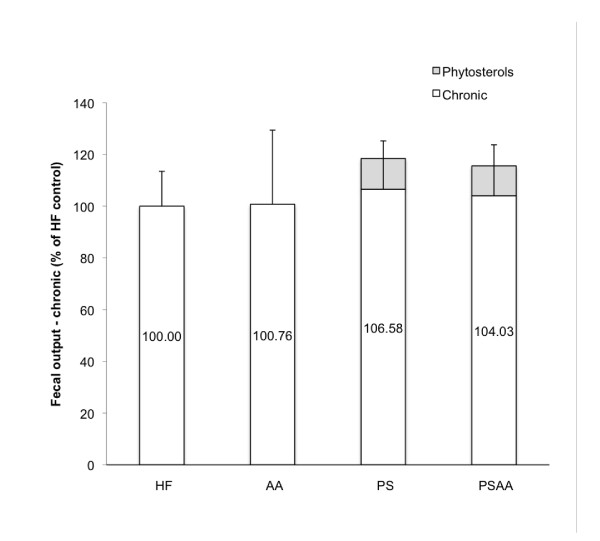
**Fecal output after 17 weeks of chronic exposure to a high fat diet with or without phytosterol or ascorbic acid**. As phytosterols are assumed to be 100% excreted, fecal output for animals in the PS and PSAA groups were corrected to account for the 1% of dietary intake of non-digestible phytosterol (PS accounts for ~ 10% of fecal output, based on a daily consumption of 1% of ~3 g chow and average daily fecal output of ~300 mg). Dietary supplements did not result in a significant difference in fecal output when compared to the high fat control diet (n = 7; ANOVA followed by a post-hoc Dunnett's Method).

**Figure 8:**
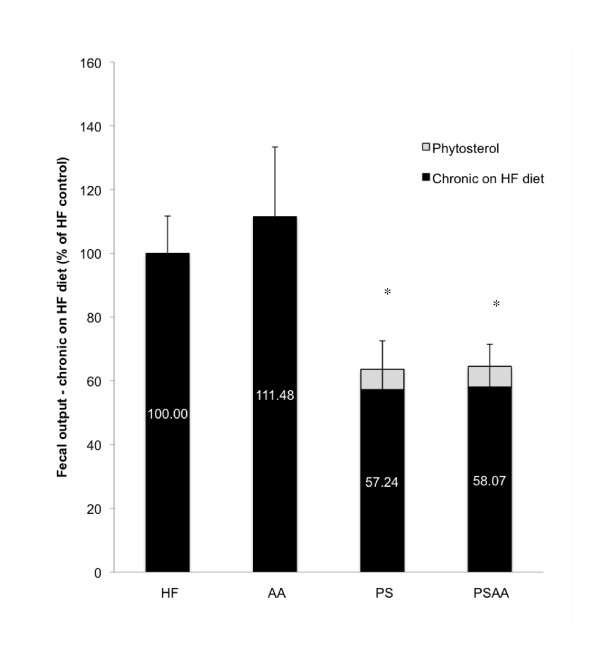
**Fecal output from animals switched to a high fat diet for 72 hours after 17 weeks of chronic exposure to a high fat diet with or without phytosterol or ascorbic acid**. As phytosterols are assumed to be 100% excreted, fecal output for animals in the PS and PSAA groups were corrected to account for the 1% of dietary intake of non-digestible phytosterol (PS accounts for ~ 10% of fecal output, based on a daily consumption of 1% of 3g chow and average daily fecal output of 300 mg). Chronic exposure to phytosterol supplements in the diet resulted in a significant decrease in fecal output when additives were withdrawn (n = 7; ANOVA followed by a post-hoc Dunnett's Method).

Animals exposed to the experimental diets with supplements for 17 weeks showed no significant differences in fecal output when compared to the HF control (n = 7, ANOVA; Figure 7). When the dietary supplements were removed for 72 hours and animals were fed a HF diet, a significant decrease in fecal output (>40%) was observed in the PS and PSAA groups (ANOVA followed by a post-hoc Dunnett's Method, Figure 8).

## Discussion

### Mass accumulation and fecal energy loss

Our data indicate that in a diet-induced obesity C57Bl/6 mouse model, chronic administration of a high fat diet with 1% w/w phytosterol and 1% w/w ascorbic acid (PSAA) results in decreased mass accumulation when compared to either the HF control diet, diets supplemented with 1% w/w ascorbic acid (AA), or diets supplemented with 1% phytosterol (PS) alone (Figure 1a; the decrease in PSAA mass accumulation was significant from weeks 7-12). The reduced rate of mass gain is not accompanied by decreased food or water intake, differences in resting metabolic rate, or alteration of maximal oxygen consumption (VO_2max_) when compared to HF control animals (data not shown; n = 8 for each dietary group; ANOVA). Interestingly, the decrease in mass accumulation is not as profound as that observed in our previous studies where the high fat diet was supplemented with 2% w/w phytostanol esterified with ascorbic acid (DAPP; Figure 1b), suggesting that esterification of the components potentiates the mass loss mechanism [17]. The mean difference in absolute mass between PSAA and HF control for the six-week period is 10.05%, and the mean difference in mass accumulation is 21.6% (i.e. the PSAA group gained on average 21% less mass each week from weeks 7-12 than the HF control group). If we look at our 2007 DAPP study data for the comparable time period, the mean difference in absolute mass between the 2% DAPP and HF control was 41%, while the mean difference in mass accumulation between the two groups for weeks 7-12 was 67.9%. In animals on the 2% DAPP-supplemented diet, this reduction in mass accumulation was correlated with adipose tissue reduction. Magnetic resonance spectroscopy and imaging studies of the mice at week 16 of the current study revealed that animals in the PS and PSAA groups experienced a slight but significant decline in adipose tissue mass when compared to the HF control (Figure 2), but only the PSAA group exhibited an accompanying reduction in mass accumulation. Our data are in concurrence with Ebine et al (2006), where supplementation with 2% w/w DAPP resulted in a significant reduction in mass accumulation in hamsters, whereas supplementation with 1% w/w unesterified phytostanol alone did not alter mass accumulation [16].

Ebine and colleagues also indicated that fecal energy output (adjusted for the phytostanol component) was not significantly different in animals administered a phytostanol-supplemented diet when compared to the control diet [16]. Our findings indicate that within both the acute (72 hour) and chronic (17 weeks) exposure experiments, supplementation with PS, AA or PSAA did not result in a significant alteration of fecal caloric content within each exposure experiment when compared to the HF control (Figure 3). However, a comparison of fecal energy output from acute exposure *vs*. chronic exposure within the *same *dietary groups revealed a significant decrease in fecal energy after 17 weeks of exposure to the HF control, PS and AA diets. A non-significant decrease in fecal caloric content was also noted in the PSAA group (t-test; p = 0.09). These data indicate that long term exposure to a high fat diet, regardless of supplement, results in a decrease in fecal energy and suggests that the animals are absorbing a greater portion of the dietary caloric content with chronic HF exposure [27]. Chronic exposure to a high fat diet has been shown to enhance intestinal cell proliferation, height of intestinal villi and to increase enterocytic migration rate from crypt to villus [28-30]. The reduced rate of mass accumulation in the PSAA group may be partly due to amelioration of the increased absorptive capacity that is associated with chronic dietary HF exposure.

### Food transit time and fecal output

Long-term exposure to dietary PS supplementation resulted in significant changes in energy assimilation and digestive efficiency. In the 72-hour exposure experiment, acute exposure to dietary supplements did not alter food transit time (Figure 4a), but did result in an increased fecal output in all groups when compared to the HF control (Figure 6). Taken together, these data suggest that short-term exposure to the dietary supplements results in some degree of interference with dietary uptake, as evidenced by a greater fecal mass with no alteration in passage rate (data are summarized in Table 1). Acute dietary PS, AA or PSAA supplementation did not alter the caloric content of the feces when compared to the HF control (Figure 3, data corrected for estimated fecal phytosterol output; n = 8, ANOVA), therefore the net effect of acute exposure on the absorptive process appears to be an overall decrease in energy assimilation of a HF diet when it is accompanied by PS, AA or PSAA. In animals chronically exposed to the HF diet with supplements, neither the fecal output (Figure 6), nor the caloric content of the feces (Figure 3) differed from the HF control, suggesting that the animals have accommodated the effects of the supplements over the course of the chronic exposure (n = 7; ANOVA).

**Table 1:** Summary of findings outlining significant differences in food transit time and fecal output for mice exposed to a HF diet with or without phytosterol or ascorbic acid supplement for 72 hours (ACUTE) or a 17-20 week exposure (CHRONIC)

	HF		AA		PS		PSAA	
	**FOOD TRANSIT TIME**	**FECAL OUTPUT**	**FOOD TRANSIT TIME**	**FECAL OUTPUT**	**FOOD TRANSIT TIME**	**FECAL OUTPUT**	**FOOD TRANSIT TIME**	**FECAL OUTPUT**

**ACUTE to HF control**	n/a	n/a	NSD	↑	NSD	↑	NSD	↑

**CHRONIC to HF control**	Did not test	n/a	Did not test	NSD	Did not test	NSD	Did not test	NSD

**CHRONIC on HF to HF control**	n/a	n/a	NSD	NSD	↓	↓	↓	↓

**CHRONIC on HF to ACUTE (paired)**	NSD	NSD	NSD	NSD	↓	↓	↓	↓

Animals in the chronic exposure group were switched to a HF control diet for 72 hours; these animals are referred to as "CHRONIC on HF". Dietary groups with supplements were compared to the HF control within each exposure experiment and assessed for statistically significant differences. To evaluate long-term changes to digestive efficiency, all dietary groups in the "CHRONIC on HF" exposure were compared values obtained from animals acutely exposed to the same dietary groups ("ACUTE"). HF control comparisons to HF control are deemed not applicable (n/a); arrows indicate values are statistically lower or higher than HF control or dietary pair group. NSD = non-significant difference.

We hypothesize that in the chronic exposure animals, increased fecal output and thus decreased energy uptake stimulates intestinal remodeling to compensate for the energy. When supplements were removed from the diet and all animals were placed on the HF control diet for 72 hours, no significant difference in the food transit time of HF control or AA supplemented diet groups was observed, indicating that chronic exposure to 1% AA supplementation did not induce changes in the intestine that would result in altered passage rate (Figure 7a). Interestingly, animals chronically exposed to diets containing PS or PSAA exhibited a significant and profound decrease in the transit time of a HF diet (Figure 7b). In the same animals, fecal output dropped by more than 40% (Figure 8). In an animal chronically exposed to PS supplements, these data describe a situation whereby the HF diet is in contact with the intestinal absorptive surface for significantly less time, yet a significantly greater proportion of the mass of the meal is absorbed when compared to acute exposure, as evidenced by a reduction in fecal output. These findings suggest that chronic exposure to PS in the diet alters the intestine and results in upregulation of its absorptive capacity. The current opinion in the literature on the mechanism of phytosterol's hypocholesterolemic properties suggests that physical interference of cholesterol absorption through either competitive positioning in the micelle, disturbance of micellar formation, or a combination of both plays a major role [10,31-33]. This mode of action may also be responsible for alteration of lipid uptake and result in a proliferation of intestinal transporters to counteract the effect of dietary phytosterol on energy procurement. When PS supplementation is removed from the diet, a significant and profound reduction in fecal output and a decrease in transit time may be partly explained by a potential increase in surface area and elevation of uptake mechanisms in the intestinal tract. Further studies involving histological and immunohistochemical analysis of the intestinal tract would be necessary to test this hypothesis.

There are a number of studies reporting that animals on a high fat diet supplemented with high doses of ascorbic acid accumulate significantly less adipose tissue than their non-supplemented counterparts [20,21]. This finding is not unique to ascorbic acid, but is also observed in response to other antioxidants. In an obese hamster model, co-administration of a high fat diet with Extramel microgranules (a melon juice extract coated with palm oil and rich in antioxidants and particularly superoxide dismutase) prevented obesity in the high fat-fed hamsters by decreasing body weight, abdominal fat, triglyceridemia, insulinemia, insulin resistance, liver lipids, and nonalcoholic steatohepatitis and preventing adipokine imbalance [34]. It is also thought that the antioxidant properties of the Mediterranean diet contribute significantly to its associated decreased risk of obesity and reduced levels of metabolic syndrome when compared to an isocaloric Western diet [35]. Several studies have reported a significant inverse relationship between plasma vitamin C concentrations and degree of obesity, further supporting a correlation between adipose tissue accumulation and ascorbic acid supplementation [22,36,37]. Our data did not support the findings of Campion et al, as the 1% AA supplementation did not result in a decrease in mass accumulation in DIO mice [20,38]. However, the potentiation of AA absorption and thus higher circulating plasma levels may have been affected by the presence of PS in the diet and its possible effect on the absorptive capacity of the intestine, which could lead to the observed increased efficacy of the PSAA combination. Esterification of ascorbic acid at position 2 protects vitamin C from destruction by oxidation and may lead to even higher circulating plasma levels, which may assist in elucidating the mechanism associated with the esterified ascorbic acid/phytostanol compound [39,40].

In our previous study using the dietary supplement disodium ascorbyl phytostanyl phosphate (DAPP), we observed that animals on a high fat diet with supplement exhibited a significant dose-dependent reduction in mass accumulation over time when compared to the HF control group [17]. In the current study, the unesterified components of DAPP (ascorbic acid and a phytosterol/phytostanol mixture) were administered separately and in combination in the presence of a high fat diet. Although the combination of supplements resulted in a significant decrease in mass loss when compared to the high fat diet alone or single-additive supplementation, the decrease was not as profound as that observed in the DAPP study. The current study did not use the same species of phytostanols that were employed in the manufacture of DAPP; therefore we cannot make a direct comparison of esterified phytostanyl *vs *unesterified phytostanol in the presence of ascorbic acid. In addition, the phosphodiester bond may afford the compound greater protection during the acid-labile digestive process and allow for absorption in a more efficacious form. However, the data support the hypothesis of decreased mass loss associated with dietary supplementation with plant sterols and antioxidants, and provide some insight into possible mechanisms of these compounds on energy assimilation in the development of obesity in high fat fed mice.

## Competing interests

The authors declare that they have no competing interests.

## Authors' contributions

SJT designed the experiments, carried out the studies, completed the data analysis and wrote the manuscript. ITYW carried out the studies and completed the data analysis. RN carried out the studies and completed the data analysis. PK carried out the studies and completed the data analysis. KWM designed the experiments, completed the data analysis and revised the manuscript. All authors have read and approved the final manuscript.
